# Supplementary material for: Host genetic selection for cold tolerance shapes microbiome composition and modulates its response to temperature
Source: eLife. 2018 Nov 20;7:e36398. doi: 10.7554/eLife.36398 (PMC6277203; doi:10.7554/eLife.36398)
Supplement: Supplementary file 1. [file elife-36398-supp1.docx]

**Host genetic selection for cold tolerance shapes microbiome composition and modulates its response to temperature**

**Fotini Kokou^1,2^, Goor Sasson^1^, Tali Nitzan^2^, Adi Doron-Faigenboim^3^, Sheenan Harpaz^2^, Avner Cnaani^2^, Itzhak Mizrahi^1*^**

^1^Department of Life Sciences, Ben-Gurion University of the Negev, Beer-Sheva, Israel; ^2^Department of Poultry and Aquaculture, Institute of Animal Sciences, Agricultural Research Organization, Rishon LeZion, Israel; ^3^Department of Vegetable and Field Crops, Institute of Plant Science, Agricultural Research Organization, Rishon LeZion Israel

*Corresponding author: Prof. Itzhak Mizrahi, Department of Life Sciences, Ben-Gurion University of the Negev, Marcus Family Campus, Beer-Sheva, Israel. Email. [imizrahi@bgu.ac.il](mailto:imizrahi@bgu.ac.il); Tel. (+972) 8 647 9836.

**Supplementary Information**

**Supplementary Tables**

| **Supplementary Table S1** \| Mean abundance (%) of the significantly affected taxa, at different taxonomic levels, due to cold exposure as indicated by Kruskal–Wallis nonparametric test. | | | | | | | |
| --- | --- | --- | --- | --- | --- | --- | --- |
|  | **Test statistic** | | ***P*-value** | **FDR**  **q-value** | **Bonferroni**  ***P*-value** | **Mean abundance**  **24^o^C** | **Mean abundance**  **12^o^C** |
| **Phyla** |  | |  |  |  |  |  |
| *TM6* | 44.74 | | 2.24E-11 | 6.95E-10 | 6.95E-10 | 0.078 | 0.0 |
| *Planctomycetes* | 28.60 | | 8.87E-08 | 9.16E-07 | 2.75E-06 | 0.286 | 0.013 |
| *Bacteroidetes* | 26.69 | | 2.38E-07 | 1.48E-06 | 7.39E-06 | 8.017 | 2.971 |
| *Verrucomicrobia* | 19.79 | | 8.61E-06 | 3.81E-05 | 0.000267 | 0.379 | 0.104 |
| **Class** |  | |  |  |  |  |  |
| *Bacilli* | 46.00 | | 1.18E-11 | 9.43E-10 | 9.43E-10 | 1.974 | 0.320 |
| *Planctomycetia* | 37.84 | | 7.65E-10 | 2.04E-08 | 6.12E-08 | 0.2829 | 0.733 |
| *Chlamydiia* | 27.56 | | 1.52E-07 | 2.43E-06 | 1.21E-05 | 0.107 | 0.011 |
| *Verrucomicrobiae* | 20.21 | | 6.92E-06 | 7.90E-05 | 1.21E-05 | 0.359 | 0.077 |
| *Erysipelotrichi* | 15.02 | | 0.000106 | 0.000933 | 0.000553 | 0.112 | 0.013 |
| *Cytophagia* | 13.25 | | 0.000272 | 0.00198 | 0.021777 | 0.044 | 0.025 |
| **Order** |  | |  |  |  |  |  |
| *Unknown order of class SJA-4 (TM6)* | 41.78 | | 1.02E-10 | 1.04E-08 | 1.47E-08 | 0.077 | 0 |
| *Bacillales* | 32.00 | | 1.54E-08 | 7.40E-07 | 2.22E-06 | 0.7722 | 0.211 |
| *Actinomycetales* | 27.74 | | 1.38E-07 | 3.98E-06 | 1.99E-06 | 3.377 | 0.567 |
| *Vibrionales* | 24.83 | | 6.24E-07 | 1.28E-05 | 8.98E-05 | 0.981 | 10.927 |
| *Alteromonadales* | 21.26 | | 4.00E-06 | 6.40E-05 | 0.000576 | 0.319 | 1.48 |
| *Rickettsiales* | 18.06 | | 2.13E-05 | 0.000279 | 0.003074 | 0.166 | 0.061 |
| *Erysipelotrichales* | 15.02 | | 0.000106 | 0.001178 | 0.015311 | 0.112 | 0.013 |
| *Cytophagales* | 13.25 | | 0.000272 | 0.002613 | 0.039199 | 0.044 | 0.025 |
| **Family** |  | |  |  |  |  |  |
| *Mycobacteriaceae* | | 47.53 | 5.41E-12 | 1.53E-09 | 1.53E-09 | 2.2424 | 0.0393 |
| *Unknown family of class SJA-4 (TM6)* | | 41.78 | 1.02E-10 | 9.62E-09 | 2.89E-08 | 0.077 | 0 |
| *Bacteroidaceae* | | 30.56 | 3.23E-08 | 1.75E-06 | 9.14E-06 | 5.2699 | 2.05 |
| *Gemmataceae* | | 29.37 | 5.97E-08 | 2.41E-06 | 1.69E-05 | 0.1823 | 5.15 |
| *Unknown family of Vibrionales order* | | 21.28 | 3.97E-06 | 0.000125 | 0.001122 | 0.9361 | 9.68 |
| *Verrucomicrobiaceae* | | 20.21 | 6.92E-06 | 0.000178 | 0.001957 | 0.359 | 0.077 |
| *Ruminococcaceae* | | 17.96 | 2.24E-05 | 0.000489 | 0.006352 | 1.296 | 0.304 |
| *Porphyromonadaceae* | | 17.15 | 3.44E-05 | 0.00065 | 0.009743 | 0.233 | 0.040 |
| *Christensenellaceae* | | 15.56 | 7.96E-05 | 0.001312 | 0.02254 | 1.125 | 0.039 |
| *Erysipelotrichaceae* | | 15.02 | 0.000106 | 0.001584 | 0.03009 | 0.112 | 0.013 |
| **Genus** | |  |  |  |  |  |  |
| *Mycobacterium* | | 47.53 | 5.41E-12 | 3.09E-09 | 3.09E-09 | 2.242 | 0.039 |
| *Turicibacter* | | 41.11 | 1.44E-10 | 2.74E-08 | 8.21E-09 | 0.621 | 0.011 |
| *Unknown genus of Parachlamydiaceae family* | | 25.79 | 3.79E-07 | 4.33E-05 | 0.000216 | 0.0848 | 0.005 |
| *Unknown genus of Ruminococcaceae I* | | 20.39 | 6.31E-06 | 0.000456 | 0.003604 | 0.031 | 0.004 |
| *Unknown genus of Gemmataceae* | | 19.27 | 1.13E-05 | 0.000456 | 0.006449 | 0.030 | 0 |
| *Unknown genus of Thermoactinomycetaceae* | | 18.98 | 1.32E-05 | 0.000684 | 0.007529 | 0.075 | 8.23E-04 |
| *Porphyromonas* | | 16.83 | 4.08E-05 | 0.001792 | 0.023293 | 0.159 | 0.021 |
| *Akkermansia* | | 16.34 | 5.27E-05 | 0.001914 | 0.30099 | 0.165 | 0.051 |
| *Unknown genus of Ruminocococcaceae II* | | 16.05 | 6.15E-05 | 0.002066 | 0.035124 | 0.235 | 0.248 |
| *Unknown genus of Isosphaeraceae* | | 15.40 | 8.68E-05 | 0.002608 | 0.049556 | 0.073 | 0.007 |
| **Exact sequence variant** | | | |  |  |  |  |
| *Mycobacterium sp.* | | 47.80 | 4.71E-12 | 1.45E-08 | 1.45E-08 | 0.913 | 0.0111 |
| *Turicibacter sp.* | | 39.38 | 3.47E-10 | 5.34E-07 | 1.07E-06 | 0.614 | 0.006 |
| *Clostridium ruminantium* | | 33.64 | 6.61E-09 | 6.77E-06 | 2.03E-05 | 0.453 | 0.004 |
| *Shewanella sp.* | | 21.69 | 3.20E-06 | 0.002121 | 0.009837 | 0.001704 | 0 |
| *Unknown species of the order Vibrionales* | | 21.54 | 3.45E-06 | 0.002121 | 0.010607 | 0.208 | 1.276 |
| *Bacteroides sp.* | | 21.12 | 4.31E-06 | 0.002121 | 0.01326 | 0.935 | 9.677 |
| *Unknown species of the family Legionellaceae* | | 19.73 | 8.91E-06 | 0.003911 | 0.027378 | 0.301 | 0.026 |
|  | | 19.27 |  | 0.00434 | 0.034718 | 0.112 | 0 |
| FDR, false discovery rate | | | | | | | |

| **Supplementary Table S2**\| Individuals with the 10 lowest and 10 highest Shannon H’ diversity in the posterior gut during warm and cold conditions from both groups (resistant and sensitive; 5 from each group). | | | |
| --- | --- | --- | --- |
| *Warm* | *Shannon H’* | *Cold* | *Shannon H’* |
| **Low Shannon H’ Index** | | | |
| Fish 2, Resistant Family2 | 1.88 | Fish 4, Resistant Family1 | 1.31 |
| Fish 2, Sensitive Family3 | 2.06 | Fish 5, Sensitive Family1 | 1.55 |
| Fish 7, Resistant Family1 | 2.84 | Fish 2, Sensitive Family2 | 1.67 |
| Fish 3, Resistant Family1 | 2.88 | Fish 7, Resistant Family1 | 1.67 |
| Fish 5, Sensitive Family3 | 2.89 | Fish 1, Resistant Family1 | 1.71 |
| Fish 4, Sensitive Family3 | 2.96 | Fish3, Sensitive Family3 | 1.73 |
| Fish 1, Resistant Family3 | 2.96 | Fish 2, Resistant Family1 | 1.79 |
| Fish 1, Resistant Family2 | 2.98 | Fish 1, Sensitive Family1 | 2.61 |
| Fish 5, Sensitive Family2 | 3.00 | Fish 7, Sensitive Family2 | 2.62 |
| **High Shannon H’ Index** | | | |
| Fish 2, Sensitive Family2 | 5.51 | Fish 3, Sensitive Family1 | 4.16 |
| Fish 1, Sensitive Family2 | 5.51 | Fish 6, Resistant Family1 | 3.82 |
| Fish 3, Sensitive Family2 | 5.41 | Fish 6, Sensitive Family2 | 3.75 |
| Fish 4, Sensitive Family2 | 5.34 | Fish 4, Resistant Family1 | 3.71 |
| Fish 4, Resistant Family1 | 5.00 | Fish 5, Resistant Family3 | 3.59 |
| Fish 6, Sensitive Family1 | 4.71 | Fish 1, Resistant Family2 | 3.50 |
| Fish 6, Resistant Family3 | 4.36 | Fish 2, Resistant Family3 | 3.46 |
| Fish 6, Resistant Family2 | 4.15 | Fish 7, Sensitive Family3 | 3.43 |
| Fish 7, Resistant Family2 | 4.12 | Fish 7, Resistant Family3 | 3.23 |
| Fish 5, Resistant Family1 | 4.09 | Fish 5, Resistant Family3 | 3.08 |

| **Supplementary Table S3** \| Two-way PERMANOVA results for selected individuals with the lowest and highest Shannon H’ index in the posterior gut (see Table S2) based on Bray–Curtis similarity. | | | | | |  |
| --- | --- | --- | --- | --- | --- | --- |
|  | **d.f.** | **SS** | **MS** | **PseudoF** | ***P*-value** | |
| *Low Diversity Microbiomes* | | | | | | |
| Temperature | 1 | 10.867 | 10.867 | 75.317 | 0.0003* | |
| Tolerance | 1 | 0.39837 | 0.39837 | 27.611 | 0.0539 | |
| Interaction | 1 | 0.49491 | 0.49491 | 34.301 | 0.0312* | |
| Residual | 16 | 23.085 | 0.14428 | 75.317 |  | |
| Total | 19 | 42.885 |  |  |  | |
|  |  |  |  |  |  | |
| *High Diversity Microbiomes* | | | | | | |
| Temperature | 1 | 0.4044 | 0.66455 | 4.2593 | 0.0016* | |
| Tolerance | 1 | 19.4109 | 0.43908 | 2.8142 | 0.0266* | |
| Interaction | 1 | 24.8219 | 0.35736 | 2.2904 | 0.0472* | |
| Residual | 16 | 2.4964 | 0.15602 |  |  | |
| Total | 19 | 3.9574 |  |  |  | |

*Statistical significance at *P* < 0.05. Permutations n = 999.

d.f., degrees of freedom; SS, sum of squares; MS, mean sum of squares.

| **Supplementary Table S4** \| Two-way Permanova results for selected individuals with the lowest and highest Shannon H’ index in the posterior gut (see Table S2) based on Euclidean distances. | | | | | |  |
| --- | --- | --- | --- | --- | --- | --- |
|  | **d.f.** | **SS** | **MS** | **PseudoF** | ***P*-value** | |
| *Low Diversity Microbiomes* | | | | | | |
| Temperature | 1 | 3.63E+11 | 3.63E+11 | 9.141 | 0.0003* | |
| Tolerance | 1 | 1.77E+11 | 1.77E+11 | 44.556 | 0.018* | |
| Interaction | 1 | 2.06E+11 | 2.06E+11 | 52.008 | 0.0115* | |
| Residual | 16 | 6.35E+11 | 3.97E+10 | 9.141 |  | |
| Total | 19 | 1.38E+12 |  |  |  | |
|  |  |  |  |  |  | |
| *High Diversity Microbiomes* | | | | | | |
| Temperature | 1 | 5.09E+10 | 5.09E+10 | 29.742 | 0.012* | |
| Tolerance | 1 | 3.47E+10 | 3.47E+10 | 20.276 | 0.0639 | |
| Interaction | 1 | 1.90E+10 | 1.90E+10 | 11.105 | 0.3437 | |
| Residual | 16 | 2.74E+11 | 1.71E+10 |  |  | |
| Total | 19 | 3.78E+11 |  |  |  | |

*Statistical significance at *P* < 0.05. Permutations n = 999.

d.f., degrees of freedom; SS, sum of squares; MS, mean sum of squares.

| **Supplementary Table S5**\| Host-associated (Indicator) species enriched in low and high diversity posterior gut microbiomes during cold and warm conditions (See Supplementary Table S2; Methods). | | | | |
| --- | --- | --- | --- | --- |
| ***Low Shannon Diversity***  ***(cold conditions)*** | ***Indval*** | ***P-value*** | ***Mean abundance % Resistant*** | ***Mean abundance % Sensitive*** |
| Otu6-*Vibrionales* unknown species | 0.83 | 0.028 | 68.39 | 0.3 |
| ***High Shannon Diversity***  ***(warm conditions)*** | | | | |
| Otu23-*Mycobacterium* | 0.94 | 0.018 | 13.5 | 0.074 |
| Otu29-*Clostridium ruminantium* | 0.90 | 0.029 | 1.07 | 0.12 |
| Otu2-*Cetobacterium somerae* | 0.87 | 0.039 | 15.07 | 2.30 |
| Otu144-*Rickettsiales mitochondria* unknown species | 0.08 | 0.041 | 0.10 | 0 |
| Otu52-*Planococcaceae* unknown species | 0.75 | 0.045 | 0.74 | 0.04 |
| Otu47-*Prevotella sp.* | 1 | 0.005 | 0 | 1.62 |
| Otu92-*Gallibacterium genomosp.* | 1 | 0.011 | 0 | 0.58 |
| Otu103-*Porphyromonas endodontalis* | 1 | 0.015 | 0 | 0.58 |
| Otu127-*Ruminococcus sp.* | 1 | 0.008 | 0 | 0.50 |
| Otu34-*Clostridiaceae* unknown species | 0.97 | 0.023 | 0.08 | 3.26 |
| Otu55-*Paraprevotellaceae* unknown species | 0.96 | 0.008 | 0.05 | 1.39 |
| Otu26-*Christensenellaceae unknown species* | 0.96 | 0.029 | 0.29 | 7.06 |
| Otu39-*Lachnospiraceae unknown species* | 0.92 | 0.010 | 0.07 | 0.94 |
| Otu38-*Bacteroides sp.* | 0.92 | 0.019 | 0.07 | 0.95 |
| Otu21-*Succinivibrionaceae unknown species* | 0.82 | 0.023 | 2.09 | 10.50 |
| Out31-*Bacteroides sp.* | 0.80 | 0.021 | 0.23 | 1.04 |
| Otu61- *Christensenellaceae unknown species* | 0.8 | 0.041 | 0 | 1.30 |
| Otu81- *YRC22 sp.*[ *Paraprevotellaceae*] | 0.8 | 0.041 | 0 | 0.87 |
| *Otu97-Staphylococcus equorum* | 0.8 | 0.045 | 0 | 0.84 |
| Otu106-*Bacteroidales unknown species* | 0.8 | 0.049 | 0 | 0.32 |
| Otu250-*Lactobacillus ruminis* | 0.8 | 0.037 | 0 | 0.20 |
| Otu321- *Paraprevotellaceae* unknown species | 0.8 | 0.044 | 0 | 0.14 |
| Otu51-*Akkermansia muciniphila* | 0.8 | 0.02 | 0.16 | 0.69 |
| Otu35-*Ruminococcaceae unknown species* | 0.79 | 0.037 | 0.04 | 3.20 |
| Otu36-*Streptococcus luteciae* | 0.78 | 0.042 | 0.06 | 3.56 |
| Otu41-*Prevotella sp.* | 0.78 | 0.04 | 0.06 | 2.81 |
| Otu148-*Fusobacterium sp.* | 0.77 | 0.048 | 0.010 | 0.311 |
| Otu60-*Bacteroides fragilis* | 0.76 | 0.045 | 0.05 | 1.36 |
| Otu177-*Treponema sp.* | 0.76 | 0.038 | 0.010 | 0.23 |
| Otu173-*Campylobacter sp.* | 0.71 | 0.037 | 0.03 | 0.25 |

| **Supplementary Table S6**\| Host-associated (Indicator) species enriched in the posterior gut microbiomes of resistant and sensitive fish at all taxonomic levels (see Methods). | | | | |
| --- | --- | --- | --- | --- |
|  | ***Indval*** | ***P-value*** | ***Mean abundance % Resistant*** | ***Mean abundance % Sensitive*** |
| **Phyla** |  |  |  |  |
| *Actinobacteria* | 0.75 | 0.004 | 0.79 | 2.93 |
| *Firmicutes* | 0.69 | 0.008 | 3.95 | 8.81 |
| *Euryarchaeota* | 0.20 | 0.012 | 0.004 | 0.05 |
| *Synergistetes* | 0.19 | 0.033 | 0.005 | 0.13 |
| *Spirochaetes* | 0.18 | 0.013 | 0.002 | 0.05 |
| **Class** |  |  |  |  |
| *Gammaproteobacteria* | 0.55 | 0.042 | 58.6 | 48.4 |
| *Actinobacteria* | 0.74 | 0.004 | 0.71 | 2.86 |
| *Clostridia* | 0.69 | 0.017 | 3.12 | 7.07 |
| *Bacilli* | 0.60 | 0.024 | 0.71 | 1.68 |
| *Methanobacteria* | 0.20 | 0.018 | 0.004 | 0.04 |
| *Sphingobacteriia* | 0.20 | 0.008 | 0.002 | 0.02 |
| *Spirochaetes* | 0.19 | 0.016 | 0.001 | 0.05 |
| *Synergistia* | 0.19 | 0.031 | 0.005 | 0.13 |
| **Order** |  |  |  |  |
| *Vibrionales* | 0.53 | 0.032 | 14.1 | 6.0 |
| *Actinomycetales* | 0.74 | 0.002 | 0.71 | 2.6 |
| *Clostridiales* | 0.69 | 0.013 | 3.1 | 6.7 |
| *Methanobacteriales* | 0.20 | 0.015 | 0.004 | 0.04 |
| *Sphingobacteriales* | 0.20 | 0.006 | 0.002 | 0.02 |
| *Spirochaetales* | 0.19 | 0.005 | 0.001 | 0.04 |
| *Synergistales* | 0.19 | 0.03 | 0.005 | 0.12 |
| **Family** |  |  |  |  |
| *Unknown family of Vibrionales* | 0.55 | 0.004 | 13.7 | 3.34 |
| *Pseudoalteromonadaceae* | 0.30 | 0.024 | 1.58 | 0.035 |
| *Neisseriaceae* | 0.246 | 0.047 | 0.26 | 0.041 |
| *Clostridiaceae* | 0.636 | 0.006 | 0.53 | 1.76 |
| *Prevotellaceae* | 0.491 | 0.049 | 0.33 | 1.13 |
| *Succinivibrionaceae* | 0.443 | 0.030 | 0.33 | 1.55 |
| *Paraprevotellaceae* | 0.332 | 0.050 | 0.05 | 0.51 |
| *Staphylococcaceae* | 0.327 | 0.013 | 0.016 | 0.14 |
| *Christensenellaceae* | 0.325 | 0.014 | 0.06 | 1.20 |
| *Unknown family of Clostridiales* | 0.315 | 0.037 | 0.05 | 0.20 |
| *Porphyromonadaceae* | 0.296 | 0.043 | 0.016 | 0.22 |
| *Unknown family of Streptophyta* | 0.291 | 0.040 | 0.04 | 0.25 |
| *Pasteurellaceae* | 0.253 | 0.032 | 0.04 | 0.12 |
| *Unknown family of Bacteroidales* | 0.210 | 0.035 | 0.01 | 0.04 |
| *Synergistaceae* | 0.188 | 0.022 | 0.004 | 0.13 |
| *Methanobacteriaceae* | 0.179 | 0.039 | 0.003 | 0.04 |
| *Caulobacteraceae* | 0.168 | 0.039 | 0.004 | 0.02 |
| *Spirochaetaceae* | 0.166 | 0.014 | 0.001 | 0.05 |
| *Actinomycetaceae* | 0.162 | 0.012 | 0.001 | 0.02 |
| *Lactobacillaceae* | 0.156 | 0.034 | 0.003 | 0.03 |
| *Coxiellaceae* | 0.151 | 0.030 | 0.002 | 0.01 |
| **Genus** |  |  |  |  |
| *Unknown genus of Vibrionales* | 0.47 | 0.031 | 12.12 | 4.93 |
| *Luteolibacter* | 0.30 | 0.044 | 0.13 | 0.05 |
| *Candidatus Arthromitus* | 0.59 | 0.024 | 0.40 | 1.25 |
| *Prevotella* | 0.50 | 0.038 | 0.35 | 1.12 |
| *Unknown genus of Paraprevotellaceae* | 0.32 | 0.007 | 0.02 | 0.23 |
| *Unknown genus of Clostridiales* | 0.31 | 0.042 | 0.04 | 0.20 |
| *Staphylococcus* | 0.30 | 0.002 | 0.006 | 0.15 |
| *Unknown genus of Christensenellaceae* | 0.30 | 0.029 | 0.07 | 1.19 |
| *Unknown genus of Clostridiaceae* | 0.28 | 0.01 | 0.01 | 0.07 |
| *Gallibacterium* | 0.20 | 0.013 | 0.007 | 0.08 |
| *YRC22* | 0.19 | 0.029 | 0.014 | 0.13 |
| *Flavobacterium* | 0.19 | 0.048 | 0.01 | 0.65 |
| *Treponema* | 0.19 | 0.004 | 0.01 | 0.047 |
| *Synergistes* | 0.19 | 0.016 | 0.04 | 0.12 |
| *Unknown genus of Rickettsiales* | 0.19 | 0.018 | 0.003 | 0.07 |
| *Lactobacillus* | 0.16 | 0.022 | 0.002 | 0.032 |
| *Parabacteroides* | 0.16 | 0.026 | 0.005 | 0.08 |
| *Unknown genus of Sphingobacteriales* | 0.13 | 0.023 | 0.001 | 0.003 |

| **Supplementary Table S7** \| Overview of the obtained RNA-Seq data in the different samples. | | | | | | |
| --- | --- | --- | --- | --- | --- | --- |
| **Sample ID** | **Temperature** | **Family** | **#Raw reads** | **#Clean reads** | **%Clean reads** | **%Mapping** |
| 12_480_L | 12 | 480 | 33,409,959 | 31873706 | 95.40181118 | 66.27 |
| 12_480_L | 12 | 480 | 18,883,215 | 17792851 | 94.22575022 | 69.07 |
| 12_480_L* | 12 | 480 | 13,215,778 | 4271760 | 32.32318218 | 6.73 |
| 12_740_L | 12 | 740 | 30,480,336 | 29356926 | 96.31431228 | 68.02 |
| 12_740_L | 12 | 740 | 18,992,107 | 17662790 | 93.00068707 | 72.42 |
| 12_740_L | 12 | 740 | 20,581,738 | 16581558 | 80.56442075 | 66.89 |
| 24_480_L | 24 | 480 | 21,384,504 | 20592540 | 96.29655193 | 74.86 |
| 24_480_L | 24 | 480 | 15,567,236 | 14892073 | 95.66292308 | 74.95 |
| 24_480_L | 24 | 480 | 13,161,987 | 12042406 | 91.49382992 | 71.78 |
| 24_740_L | 24 | 740 | 16,404,872 | 15499596 | 94.48166374 | 79.15 |
| 24_740_L | 24 | 740 | 12,957,265 | 11866150 | 91.57912569 | 52.45 |
| 24_740_L | 24 | 740 | 12,720,316 | 11620267 | 91.35203088 | 70.78 |
| *This sample was excluded from further analysis. | | | | | | |
